# Effectiveness of a Smoking Cessation Program during the COVID-19 Pandemic

**DOI:** 10.3390/healthcare11111536

**Published:** 2023-05-24

**Authors:** Aleksandra Kruk, Celina Czerwińska, Justyna Dolna-Michno, Elżbieta Broniatowska, Emanuel Kolanko

**Affiliations:** 1 Department of Pulmonology, John Paul II Hospital, 31-202 Kraków, Poland; 2 Faculty of Medicine and Health Sciences, Andrzej Frycz Modrzewski Krakow University, 30-705 Kraków, Poland

**Keywords:** smoking cessation program, COVID-19, interventions, Poland

## Abstract

The coronavirus disease-2019 pandemic has caused major obstacles for effective smoking cessation programs by significantly limiting access to healthcare. This cross-sectional analysis aimed to assess the effectiveness of a self-developed smoking cessation program during the pandemic. The program was based on remote lectures, educational interventions, and hybrid services provided by an outpatient clinic. We assessed 337 participants enrolled to the program between January 2019 and February 2022. Data on demographic characteristics, medical history, and smoking status at baseline and after at least 1-year follow-up were collected from medical records and a standardized self-developed questionnaire. Participants were classified into two groups according to their current smoking status. The smoking cessation rate at 1 year was 37% (95% confidence interval [CI]: 31–42%). Major predictors of smoking cessation were the place of residence, ability to refrain from smoking during severe illness, and the number of cigarettes smoked per day. The proportion of participants with high levels of nicotine dependence at baseline was 40.8% (95% CI: 34.5–47.5%) vs. 29.1% (95% CI: 23.4–35.5%) after the program. In the group that did not quit smoking, there were more participants who smoked within 5 min after waking up than before the program (40.4% [95% CI: 34.0–47.1%] vs. 25.4% [95% CI: 19.9–31.6%]). Effective smoking cessation interventions can be performed using remote counseling and education.

## 1. Introduction

Nicotine dependence remains one of the major public health issues [1]. According to the World Health Organization (WHO) report, there are 1.3 billion regular smokers in the world [2]. The highest prevalence of smoking was noted in Europe, particularly in Eastern Europe (25% and 28% of the population, respectively) [3]. Among European countries, the highest percentage of smokers was reported in Greece (42%) and the lowest—in Sweden (7%) [4]. In Poland, numerous public campaigns and tobacco control measures have resulted in a significant decline in the prevalence of smoking and smoking-related mortality rates since 1990 [5]. Nevertheless, the prevalence of cigarette smoking in Poland remains high and is currently estimated at 28% (30.8% in men and 27.1% in women) [3].

Numerous studies reported tobacco use to be a major cause of death and disability [6]. Tobacco contains nicotine that is involved in developing neural adaptations and psychological mechanisms leading to addiction. While nicotine alone is quite harmless, the substances contained in tobacco smoke, such as carcinogens, toxicants, particulate matter, and carbon monoxide, are dangerous to health [7]. Repeated exposure to tobacco smoke is a well-established risk factor for coronary artery disease, chronic obstructive pulmonary disease, and death [8].

There have been numerous campaigns and guidelines aimed at reducing the prevalence of smoking and preventing negative health outcomes among smokers [9]. In addition, governments introduce tobacco control policies, such as taxation of tobacco products, prohibiting tobacco smoking in certain spaces, and raising public awareness through mass media campaigns and warning labels on tobacco products [10]. Moreover, the medical community stresses the need to individualize medical interventions and therapeutic strategies in smokers and to recognize nicotine dependence as a disease, with the development of smoking cessation guidelines and quality standards for tobacco cessation specialists and outpatient tobacco cessation services [11]. It is generally recommended that healthcare professionals should regularly identify active smokers and include this information in their medical records [12]. Moreover, adherence to established tobacco treatment protocols is recommended. A trained health professional should ask the patient whether he or she is an active smoker, advice smoking cessation, assess the patient’s readiness to quit smoking, assist in addiction treatment by providing necessary therapy or medication, and monitor and support abstinence during follow-up visits [11]. Available treatment options include pharmacotherapy and behavioral support [13].

Currently, bupropion and cytosine are registered by the Food and Drug Administration and European Medicine Agency to treat nicotine dependence. Moreover, various nicotine replacement therapy products are available over the counter, such as gums, lozenges, patches, inhalers, and nasal sprays [14]. Multiple studies worldwide confirmed that the most effective strategy in tobacco cessation treatment is to combine medication use with behavioral counseling [15]. Psychotherapy should be aimed at raising smokers’ awareness of their smoking patterns and identifying smoking triggers [16]. Moreover, it attempts to modify patients’ thoughts and emotions linked to smoking and provides motivation, support, and guidance on coping with urges to smoke [17]. It is recommended that tobacco dependence treatment is provided both by primary care physicians and by psychiatrists and therapists in a specialist outpatient clinic. However, access to such a specialized treatment in the outpatient setting is very limited due to financial and organizational reasons [14].

The effectiveness of smoking cessation programs is assessed mainly on the basis of self-reported information collected from patients at specific time points after they have completed the program. A systematic review of studies exploring different cessation methods revealed that abstinence rates decreased with time. For example, in one cohort study included in the review, the quit rate was 88.2% at 4 weeks, 54% at 6 months, and only 36% at 12 months after tobacco cessation [18].

The coronavirus disease-2019 (COVID-19) pandemic proved to be a major obstacle to smoking cessation. Social restrictions and limited access to healthcare made it more difficult to seek professional help [19]. Numerous healthcare facilities, including outpatient clinics, were able to provide remote services only, for example, via phone. Several studies investigated changes in the use of different nicotine products caused by the COVID-19 pandemic [20]. It was reported that higher mortality from COVID-19 infection among smokers motivated many people to quit smoking [21]. On the other hand, increased stress levels and boredom during the pandemic triggered some people to smoke more frequently [22]. Interestingly, a study assessing the impact of COVID-19 on the delivery of tobacco cessation treatment for cancer patients at 34 cancer centers suggested that remote services can be as good as traditional ways of providing treatment [23]. In this study, we aimed to assess the effectiveness of a self-developed smoking cessation program for tobacco users that was based on lectures, educational interventions, and hybrid services provided at an outpatient clinic during the COVID-19 pandemic.

## 2. Materials and Methods

This study was carried out as a part of the self-developed smoking cessation program called “Take a deep breath” (in Polish, “Weź głęboki oddech”), conducted in three Polish voivodeships: Małopolskie, Świętokrzyskie, and Podkarpackie. The most important activities within the program included the launch of a tobacco treatment center at John Paul II Hospital in Kraków, Poland, establishing a cooperation between the hospital and primary care facilities with the aim to improve the care over patients with tobacco dependence, and the implementation of numerous educational interventions. The primary goal of the program was to reach the maximum number of smokers from the target areas who were willing to quit smoking and would respond positively to the proposed assistance.

### 2.1. Study Design

To assess the effectiveness of interventions implemented as part of the program, we used an observational cross-sectional survey. Smokers were actively recruited during various activities promoting smoking cessation, such as public lectures, and during hospitalization or medical visits in specialized outpatient clinics or primary care practices participating in the program. Events were widely advertised in all available media. Individuals who required professional assistance in smoking cessation, as determined on the basis of a medical interview, were enrolled. In each participant, the initial smoking status was assessed based on a standardized self-developed questionnaire (Questionnaire S1, Supplementary Materials). Then, after a minimum of 1-year participation in the program, a follow-up interview was conducted by phone, which examined the current smoking status and the effectiveness of the smoking cessation interventions used within and outside the tobacco treatment center (Questionnaire S2, Supplementary Materials). The questionnaire was developed based on the WHO guidelines [24].

We collected the following data based on medical records and the standardized questionnaire: demographic characteristics, comorbidities, smoking status at enrollment, and activities conducted as a part of smoking cessation interventions within and outside the tobacco treatment center.

Participants were classified into two groups based on their smoking status at follow-up: a group of nonsmokers including participants who quit smoking during the program, and a group of current smokers including participants who did not quit smoking. We also separately assessed participants who attended and those who did not attend our tobacco treatment center.

### 2.2. Study Population

In this study, we assessed all individuals who participated in the “Take a deep breath” program between January 2019 and February 2022. All participants were long-term smokers and were over 18 years old at enrollment. Moreover, all participants were literate and cognitively able to answer all survey questions. Exclusion criteria were the lack of consent, death during the program, and age under 18 years.

At baseline, the program included 513 participants, 289 men (56.3%) and 224 women (43.7%). A total of 176 participants were excluded from the final analysis and follow-up: 49 patients who died during the program (including 14 deaths due to lung cancer) and 20 patients who did not answer the phone or did not consent to the interview. The final study sample included 337 participants.

### 2.3. Therapy Provided at the Tobacco Treatment Center

As part of smoking cessation treatment in our center, participants were consulted by a psychiatrist for mental status assessment. They also underwent a psychological consultation to assess the level of nicotine addiction and motivation to quit smoking. Subsequently, they received an individual addiction therapy conducted by a trained addiction specialist.

Smoking cessation counseling was provided using a cognitive-behavioral model. Following psychiatric and psychological consultations, an addiction therapist developed a conceptual framework that provided the basis for an individualized therapy plan with the patient, with therapy duration and techniques adjusted to individual needs. The role of the therapist was to develop a bond with the patient, explore the patient’s complaints and establish a diagnosis, determine triggering, sustaining, susceptibility, and protective factors, develop an initial conceptualization of the problem and share it with the patient, and, finally, set therapy goals. Major strategies used included psychoeducation, monitoring of tobacco cravings, describing triggering situations, classification of tobacco cravings, personal work, and thinking through potential consequences of behavior. Other therapeutic techniques were also used, such as exaggerations or paradox, searching for an alternative proof of the correctness of a thesis, reattribution, thought stopping, distraction, activity planning, relaxation training (Jacobson’s relaxation technique, autogenic training), behavioral experiments, and problem-solving techniques. Moreover, each participant was monitored for abstinence.

Pharmacological treatment of tobacco dependence included the use of such medications as bupropion, varenicline, or cytosine, which alleviate craving symptoms and reduce the urge to smoke. Nicotine replacement therapy was administered particularly in patients who succeeded in quitting smoking to relieve symptoms of abstinence.

Importantly, before the COVID-19 pandemic, the tobacco treatment center provided in-person services. After November 2019, a hybrid form was adopted, whereby consultations and therapy were provided partly in person and partly remotely via phone.

### 2.4. Outcomes

All outcomes were recorded from the day of the patient’s enrollment in the program until the day of telephone interview. Patients who quit smoking were defined as patients who answered “Not at all” to Question 1 in Questionnaire S2 (“Are you a current smoker? Do you smoke every day, not every day, or not at all?”; Supplementary Materials). Patients who selected a different answer were defined as those who continued to smoke.

All comorbidities assessed in this study referred to diseases diagnosed before enrollment in the program and were identified on the basis of the patients’ medical records and questionnaires. Chronic obstructive pulmonary disease was defined as a heterogonous lung condition with chronic respiratory symptoms, diagnosed based on the presence of limitation to airflow that is not fully reversible and a ratio of forced expiratory volume in the first second to forced vital capacity of less than 0.7 on spirometry [25]. Lung cancer was defined as a malignant primary tumor in the lungs, including small and non-small lung cancer [26]. Obstructive apnea was defined as over five predominantly obstructive respiratory events per hour of sleep, observed on polysomnography [27]. Coronary artery disease was defined as prior myocardial infarction, coronary artery bypass grafting surgery, or obstructions in coronary arteries identified during percutaneous coronary intervention [28]. Finally, heart failure was defined as symptoms consistent with New York Heart Association functional class I–IV [29].

The interview at baseline included the questions: “How soon after waking up do you smoke your first cigarette?” and “How many cigarettes do you smoke on average per day or per week?” with the aim to assess the level of physical addiction to nicotine according to the Heaviness Smoking Index (HIS). The index was derived from the Fagerström test as its simplified version and is an internationally approved and a widely used tool for assessing nicotine dependence with efficacy comparable to that of the Fagerström Nicotine Dependence Test [30].

During the consultation in the tobacco treatment center, the complete Fagerström test was performed to further monitor the level of tobacco dependence and to differentiate between biological and behavioral addiction. Patients who score more than 7 points in the test present with symptoms of biological addiction, which is a more severe form of addiction and requires greater professional assistance to help in quitting. Patients who score less than 4 points present with less severe symptoms of addiction and are more likely to quit without additional support [31]. To assess the patient’s readiness to quit, the Schneider motivation test was performed [32]. It consists of 12 questions with the possible answer of “yes” (1 point) or “no” (0 points). Patients who score 7 points or more are considered highly motivated to quit smoking and are more likely to benefit from smoking cessation interventions.

### 2.5. Bioethics Committee Approval

The study was conducted in accordance with the Declaration of Helsinki and was approved by the Bioethics Committee of the District Medical Council in Kraków, Poland (No., OIL/KBL/5/2023).

### 2.6. Statistical Analysis

Continuous variables were presented as median and interquartile range (IQR). The normal distribution of continuous variables was verified by the Shapiro–Wilk test. The Mann–Whitney U-test was applied to compare two groups for nonnormally distributed continuous variables. The categorical (qualitative) variables were presented as the numbers and appropriate percentages. The Chi-squared test or Fisher’s exact test was used to compare categorical variables between groups. Questionnaire responses before and after the program were compared using the Wilcoxon test or the McNemar–Bowker test as appropriate. The backward stepwise multivariable logistic regression model was built to identify predictors of smoking cessation (only variables with a *p* value of less than 0.25 were selected for the model). The obtained model was adjusted for age and sex. The model’s goodness of fit was verified by the Hosmer–Lemeshow test, and the c-statistics (c-index, area under the curve) was used to assess the predictive accuracy of the model. *p* values lower than 0.05 were considered significant for the two-sided tests. The R package [33] and Statistica software (TIBCO Software Inc. (2017). Statistica (data analysis software system), version 13. http://statistica.io accessed on 1 March 2023) were used for all the analyses. 

## 3. Results

The final sample included 337 patients (183 men [54.3%] and 154 [45.7%] women). Most participants were well educated (high-school education and higher, 269 [79.8%]; preschool, primary, middle-school education, 68 [20.2%]). Most participants were employed (282 [55.2%]) and lived in big cities (271 [52.8%]). The mean age of participants at enrollment in the program was 54.8 (SD 14.9) years. There were 6 participants (1.2%) who were homeless and 36 participants (7%) with disability.

### 3.1. Demographic and Clinical Characteristics of the Study Population

Detailed demographic and clinical characteristics of the study population are presented in Table 1. In the follow-up interview, 124 participants reported abstinence from smoking and 213 reported to be smokers. The female-to-male ratio was comparable between smokers (51.5% vs. 48.5%) and quitters (47.6% vs. 52.4%).

At baseline, most participants (267 [79.2%]) reported smoking a few times a day, while 21 participants (6.2%) smoked once a day. The most common tobacco products were cigarettes (294 participants [87.2%]. The use of e-cigarettes was reported only by 22 participants (6.5%). A similar tendency was observed throughout the program.

At baseline, most participants showed a high and moderate level of nicotine dependence, as measured by the HSI (143 [42.4%] and 161 [47.8%], respectively). During the program, 37% of participants stopped smoking and their main motivation for cessation was health concerns (65%) and the onset of severe illness (42%), while financial reasons were less common (1%). Reduced smoking was reported in 18.9% of participants; 13% of participants reduced the number of cigarettes smoked but not the frequency of smoking. Detailed data on the number of cigarettes smoked per day are presented in Table 2.

At baseline, most participants were able to refrain from smoking in public places where smoking was not allowed (210 [62.3%]) and during severe illness (204 [60.5%]).

### 3.2. Analysis of Participants Who Did Not Quit Smoking after the Program

In the group who reduced smoking consumption, the number of participants with a high level of nicotine dependence decreased, while the number of participants with a moderate and low level of dependence increased after the program. This indicates that a high proportion of participants with high levels of dependence before the program reduced the quantity and frequency of smoking. Detailed data are presented in Table 3.

### 3.3. Characteristics Participants Who Attended the Tobacco Treatment Center

Of the 337 participants, 157 (46.6%) had at least one consultation in the tobacco treatment center. Twelve participants had in-person consultations only, 36 participants had remote consultations only, and 109 participants used both forms of consultation.

In the group of nonsmokers who attended the tobacco treatment center, the median duration of abstinence was 12 months (IQR, 6–18); 55 people (28.9%) maintained abstinence for a minimum of 1 year.

Importantly, volunteers for the program included mainly participants whose main motivation to quit smoking was concern about health. In contrast, among the remaining participants, who did not attend the center, the main motivation was severe illness (50%).

Most participants (75.8%) did not use any medication, mainly due to medical contraindications or lack of consent. Medication use was reported in 16.8% of participants. The most common medications are presented in Table 4.

### 3.4. Predictors of Smoking Cessation

The regression model (Table 5) revealed that the key predictors of successful smoking cessation were the place of residence, ability to refrain from smoking during severe illness, and the number of cigarettes smoked per day. Participants who reported to be able to refrain from smoking during severe illness were 1.5-fold more likely to quit smoking than those who were not able to do so. Residents of big cities were 1.5-fold more likely to quit smoking than those from small cities and villages. Finally, participants who smoked fewer than 20 cigarettes a day were twice more likely to quit smoking than those who smoked more than 20 cigarettes a day.

## 4. Discussion

The current study assessed the effectiveness of the self-developed smoking cessation program. The success rate of our program was 37%. In previous research, success rates for smoking cessation programs at 12-month follow-up ranged from 19% to 48% [34]. Moreover, those rates decreased with time from the intervention and depended on several factors, such as the type of therapy, study population, and the setting of the treatment center [34]. To increase the success rate, we focused on an individualized and multidisciplinary approach to treatment, which encompassed psychiatric and psychological consultation, counseling, psychoeducation, and pharmacotherapy, including medication. According to multiple guidelines, both counseling and medications are effective when used alone, but the best outcomes are achieved when these strategies are combined [11,35,36]. In a meta-analysis including 19,488 smokers, in which abstinence was assessed at 6-month follow-up, it was shown that every smoking cessation intervention increases the chances of quitting. A combination of medical treatment and professional behavioral therapy resulted in a quit rate of 15.2%, as compared with 8.6% with brief counselling alone [13]. However, in another three studies conducted in the setting of pulmonology clinics, the smoking cessation rates were reported to be higher and reached 41.2%, 45.5%, and 40% [37]. This could be explained by the fact that, similar to our study, the treatment centers were organized within a pulmonary clinic or hospital, and thus most participants had already developed some negative consequences of smoking and may have had a stronger motivation to quit.

It is important to note that our program was conducted during the COVID-19 pandemic. Therefore, some complications occurred, such as inability to provide group therapy, and some necessary changes had to be introduced, such as a shift to remote lectures and consultations. Moreover, participants faced some psychological issues following the outbreak of the pandemic. It was reported that higher levels of anxiety among patients may lead to increased tobacco consumption [38]. In another study, 18.5% of smokers reported lower cigarette consumption and 13.8% reported higher cigarette consumption following the pandemic [20]. One-third of smokers reported increased motivation to smoking cessation, mainly from fear of COVID-19-related complications. In line with these findings and our own results, a similar study reported that the smoking cessation rate after the COVID-19 outbreak was 31.1% vs. 23.8% before the pandemic [39].

In several previous studies assessing smoking cessation programs based on pharmacotherapy, reported quit rates were comparable to that in our study or even higher (40%, 45.3%, and 53%) [34,40,41]. In our study, there was no significant correlation between medication use and the smoking cessation rate. However, in the cited studies, medication (or placebo in the control group) was administered in all participants, and the treatment was carefully monitored, including possible side effects. In our study, we focused on providing an individualized treatment that encompassed a wide range of interventions and assessed all the outcomes. Although medication was offered to all participants, many of them had contraindications or did not consent to pharmacotherapy. The lack of consent might have been due to limited access to healthcare during the COVID-19 pandemic, and hence the fear of possible side effects may have been greater than usual.

Our study showed that in addition to patients who quit smoking, there were also many participants who managed to reduce their nicotine dependence level along with the quantity or frequency of cigarette consumption. Although this is not the primary aim of smoking cessation interventions, a meta-analysis of 51 studies showed that the strategy of reducing consumption before quitting altogether is not inferior to sudden smoking cessation [42]. In fact, it may be even more effective if combined with additional pharmacotherapy.

Although our study population was selected randomly, most participants were reported to be heavy smokers, with high HSI and Fagerström scores and often with biological dependence, a long history of smoking, and high cigarette consumption. However, the high level of nicotine dependence in our study probably results from the fact that volunteers were recruited mainly in the hospital setting and already presented with adverse health effects of smoking. In our study, the level of nicotine dependence was not a predictor of a quit rate, unlike the number of smoked cigarettes. This indicates that the baseline assessment of the number of cigarettes smoked daily may be sufficient to predict the patient’s quitting behavior, and that HSI measurement may not be necessary [43].

Our study also showed that residents of big cities are more likely to quit smoking. This may be explained by the fact that our tobacco treatment center was based in a tertiary specialist hospital in a big city and the program may have been less accessible to rural communities, especially during the COVID19 pandemic. In our study, age and sex were not predictors of successful nicotine cessation, which is in line with similar studies conducted before and during the COVID-19 pandemic [44,45,46,47,48,49].

Finally, we noted that more participants in the group who did not quit smoking began to smoke earlier in the day than before the program. However, this was not observed in the group who reduced tobacco consumption. This may be consistent with the so called “hardening hypothesis”, which says that as the prevalence of smoking in a population declines, the inclination of more heavily addicted (“hardened”) smokers to quit smoking decreases [50].

Our study has several limitations. First, as a follow-up interview with all participants was not possible (due to death, change of phone number, or lack of consent), the collected data may be incomplete. Moreover, our research is based solely on self-reported information without any diagnostic tests. Therefore, some of the data may be incorrect, because research shows that patients are not always honest about their nicotine consumption [51]. Finally, although no social group was favored or excluded or discriminated from the program, most of them were highly motivated to quit smoking or had other important incentives such as severe illness or family reasons that encouraged them to introduce healthy lifestyle changes. This may have increased the success rate of the program. Nevertheless, our results are in line with other reported research, which enhances their credibility.

## 5. Conclusions

In conclusion, our single-center experience indicates that a smoking cessation program combining remote counseling and education with face-to-face interventions is associated with similar quit rates during the COVID-19 pandemic as those before the pandemic.

## Figures and Tables

**Table 1:** Demographic and clinical characteristics of the study population.

Parameter	Overall (n = 337)	Current Smokers (n = 213)	Nonsmokers (n = 124)	*p* Value	Attended Tobacco Treatment Center (n = 190)	Did Not Attend Tobacco Treatment Center (n = 142)	*p* Value
age (y), median (IQR)	56 (43–64)	55 (44–65)	57 (38–64)	0.42	54.5 (43.0–63.8)	58.5 (42.0–67.0)	0.14
male sex, n (%)	183 (54.3)	118 (55.4)	65 (52.4)	0.6	105 (55.3)	77 (54.2)	0.85
**socioeconomical status, n (%)**							
person with disability	19 (5.6)	12 (5.6)	7 (5.6)	0.96	12 (6.3)	7 (4.9)	0.62
**employment status, n (%)**							
unemployed	30 (8.9)	19 (8.9)	11 (8.9)	0.17	9 (4.7)	21 (14.8)	<0.0001
professionally passive	106 (31.5)	69 (32.4)	37 (29.8)		45 (23.7)	57 (40.1)	
employed	201 (59.6)	125 (58.7)	76 (61.3)		136 (71.6)	64 (45.1)	
**education, n (%)**							
compulsory (pre-school, primary school, middle school)	68 (20.2)	18 (14.5)	68 (20.2)	0.048	22 (11.6)	46 (32.4)	<0.0001
not compulsory (higher than middle-school)	269 (79.8)	106 (85.5)	269 (79.8)		168 (88.4)	96 (67.6)	
**place of residence, n (%)**							
city	191 (56.7)	112 (52.6)	79 (63.7)	0.1	131 (68.9)	58 (40.8)	<0.0001
town	72 (21.4)	52 (24.4)	20 (16.1)		29 (15.3)	42 (29.6)	
country	74 (22.0)	49 (23.0)	25 (20.2)		30 (15.8)	42 (29.6)	
**comorbidities, n (%)**							
COPD	41 (12.2)	25 (11.7)	16 (12.9)	0.75	13 (6.8)	28 (19.7)	0.0004
asthma	20 (5.9)	13 (6.1)	7 (5.6)	0.86	5 (2.6)	15 (10.6)	0.003
lung cancer	15 (4.5)	7 (3.3)	8 (6.5)	0.17	5 (2.6)	10 (7.0)	0.055
sleep apnea	29 (8.6)	18 (8.5)	11 (8.9)	0.89	9 (4.7)	20 (14.1)	0.003
CAD	24 (7.1)	15 (7.0)	9 (7.3)	0.94	13 (6.8)	11 (7.7)	0.72
heart failure	14 (4.2)	7 (3.3)	7 (5.6)	0.29	7 (3.7)	7 (4.9)	0.58
diabetes mellitus	30 (8.9)	22 (10.3)	8 (6.5)	0.23	6 (3.2)	24 (16.9)	<0.0001

CAD, coronary artery disease; COPD, chronic obstructive lung disease.

**Table 2:** Data on the smoking status of participants at enrollment to the program based on Questionnaire S1.

Parameter	Overall (n = 337)	Current Smokers (n = 213)	Nonsmokers (n = 124)	*p* Value	Attended Tobacco Treatment Center (n = 190)	Did Not Attend Tobacco Treatment Center (n = 142)	*p* Value
**How soon after waking up do you smoke your first cigarette** **?**							
Within the first 5 min	85 (25.2)	58 (27.2)	27 (21.8)	0.04	65 (34.2)	20 (14.1)	0.0001
Within the first 6–30 min	99 (29.4)	66 (31.0)	33 (26.6)		52 (27.4)	45 (31.7)	
Within the first 31–60 min	55 (16.3)	34 (16.0)	21 (16.9)		31 (16.3)	20 (14.1)	
After 1 h	78 (23.1)	38 (17.8)	40 (32.3)		33 (17.4)	45 (31.7)	
**Do you find it difficult to refrain from smoking in places where it is not allowed?**							
Yes	127 (37.7)	81 (38.0)	46 (37.1)	0.56	78 (41.1)	48 (33.8)	0.21
**Number of cigarettes per day**							
10 or less	115 (34.1)	54 (25.4)	61 (49.2)	0.0003	50 (26.3)	63 (44.4)	0.002
11–20	140 (41.5)	94 (44.1)	46 (37.1)		87 (45.8)	51 (35.9)	
21–31	42 (12.5)	33 (15.5)	9 (7.3)		29 (15.3)	12 (8.5)	
31 or more	20 (5.9)	15 (7.0)	5 (4.0)		15 (7.9)	5 (3.5)	
**Ability to refrain from smoking during severe illness**							
Yes	204 (60.5)	117 (54.9)	87 (70.2)	0.027	102 (53.7)	98 (69.0)	0.001
**Most common tobacco products**							
Cigarettes	294 (87.2)	184 (86.4)	110 (88.7)	0.45	167 (87.9)	122 (85.9)	0.79
E-cigarettes	22 (6.5)	11 (5.2)	11 (8.9)		13 (6.8)	9 (6.3)	
**Frequency of smoking**							
Once a day	21 (6.2)	12 (5.6)	9 (7.3)	0.18	15 (7.9)	5 (3.5)	0.022
Several times a day	267 (79.2)	170 (79.8)	97 (78.2)		154 (81.1)	110 (77.5)	
Several times a week	11 (3.3)	6 (2.8)	5 (4.0)		7 (3.7)	4 (2.8)	
Occasionally	16 (4.7)	6 (2.8)	10 (8.1)		4 (2.1)	12 (8.5)	
Level of nicotine dependence *							
High	143 (42.4)	87 (40.8)	56 (45.2)	0.83	76 (40.0)	65 (45.8)	0.35
Moderate	161 (47.8)	100 (46.9)	61 (49.2)		98 (51.6)	62 (43.7)	
Low	13 (3.9)	9 (4.2)	4 (3.2)		16 (8.4)	15 (10.6)	
**Attended tobacco treatment center**	190 (56.4)	124 (58.2)	66 (53.2)	0.38			

Data are presented as number (percentage) of participants. * Measured by the Heaviness Smoking Index.

**Table 3:** Changes in smoking patterns in participants who did not quit smoking during the program.

Parameter	Before the Program (n = 213)	After the Program (n = 213)	*p* Value
**Difficulty in refraining from smoking in places where it is not allowed**			
Yes	81 (38.0)	77 (36.2)	0.56
**How soon after waking up do you smoke your first cigarette?**			
Within the first 5 min	54 (25.4)	86 (40.4)	0.0004
Within the first 6–30 min	94 (44.1)	82 (38.5)	
Within the first 31–60 min	33 (15.5)	33 (15.5)	
After 1 h	15 (7.0)	5 (2.3)	
**Ability to refrain from smoking during severe illness**			
Yes	117 (54.9)	149 (70.0)	0.006
**Most common tobacco products**			
Cigarettes	184 (86.4)	178 (83.6)	0.67
E-cigarettes	11 (5.2)	28 (13.1)	
**Frequency of smoking**			
Every day	182 (85.4)	190 (89.2)	0.09
Less than every day	12 (5.6)	22 (10.3)	
Level of nicotine dependence *			
High	87 (40.8)	62 (29.1)	0.003
Moderate	100 (46.9)	124 (58.2)	
Low	9 (4.2)	20 (9.4)	

Data are presented as number (percentage) of patients. * Measured by the Heaviness Smoking Index.

**Table 4:** Characteristics of participants who attended the tobacco treatment center.

Parameter	Overall (n = 190)	Current Smokers (n = 124)	Nonsmokers (n = 66)	*p* Value
**medication**				
yes	32 (16.8)	22 (17.7)	10 (15.2)	0.72
**type of medications used**				
cytisine	24 (75)	18 (81.8)	6 (60)	0.2
NRT	21 (65.6)	11 (50)	10 (100)	
varenicline	1 (3.1)	1 (4.5)	0 (0)	
**years of smoking**				
median [Q1-Q3]	25.0 [7.00–40.0]	25.0 [9.25–40.0]	24.0 [6.25–40.0]	0.54
**motivation level**				
strong	115 (60.5)	72 (58.1)	43 (65.2)	0.99
**type of addcition**				
physical	72 (37.9)	50 (40.3)	22 (33.3)	0.29

Data are presented as number (percentage) of participants unless indicated otherwise. NRT, nicotine replacement therapy.

**Table 5:** Predictors of smoking cessation in a multiple regression analysis.

Predictor	Odds Ration Per	Odds Ratio	95% CI	*p* Value
age at enrollment in the project	1 year	0.99	0.98–1.01	0.37
ability to refrain from smoking during severe illness	yes/no	1.81	1.09–3.02	0.022
diabetes	no/yes	2.30	0.91–5.78	0.076
number of cigarettes	max 20 cig. a day/more than 20 cig. a day	2.39	1.20–4.75	0.013
place of residence	big city/small city or village	1.62	1.001–2.62	0.049
sex	female/male	0.98	0.60–1.60	0.924

## Data Availability

No new data were created or analyzed in this study. Data sharing is not applicable to this article.

## Supplementary Materials

The following supporting information can be downloaded at: https://www.mdpi.com/article/10.3390/healthcare11111536/s1, Questionnaire S1: Assessment of the smoking status at baseline in participants of the “Take a deep breath” program; Questionnaire S2: Follow-up interview by phone after at least a minimum of 1-year participation in the program.
