# Supplementary material for: Effectiveness of a Smoking Cessation Program during the COVID-19 Pandemic
Source: Healthcare (Basel). 2023 May 24;11(11):1536. doi: 10.3390/healthcare11111536 (PMC10252602; doi:10.3390/healthcare11111536)
Supplement: Supplementary file 1 [file healthcare-11-01536-s001.zip › healthcare-2331075-supplementary.pdf]

SUPPLEMENTARY MATERIAL

Article title: Effectiveness of a Smoking Cessation Program during the COVID-19 Pandemic

Authors: Aleksandra Kruk, Celina Czerwińska, Justyna Dolna-Michno, Elżbieta Broniatowska and Emanuel Kolanko

Questionnaire S1 Assessment of the smoking status at baseline in participants of the "Take a deep breath" program.

**Question**	**Answer**
Q1. How soon after waking up do you smoke your first cigarette	Within the first 5 minutes<br>Within the first 6-30 minutes<br>Within the first 31-60 minutes
Q2. Do you find it difficult to refrain from smoking in places where it is not allowed?	Yes<br>No
Q3. How many cigarettes do you smoke on average per day?	10 a day or less<br>11-20 a day<br>21-30 a day<br>31 a day or more
Q4. Can you refrain from smoking even when you are sick and stay in bed most of the day?	Yes<br>No
Q5. What type of tobacco products do you use most often?	Cigarettes<br>E-cigarettes<br>Tobacco heating system<br>Water pipe<br>Pipe<br>Cigar<br>Other<br>I do not know / I do not want to answer this question
Q6. How often do you smoke?	Once a day<br>Few times a day<br>Few times a week<br>Occasionally

Questionnaire S2 Follow-up interview by phone after at least a minimum of 1-year participation in the program

**Area of assessment**		**Question**	**Answer**
Prevalence of tobacco smoking; key aspects of tobacco surveillance	Current smoking status	Q1. Are you a current smoker? Do you smoke every day, not every day, or not at all?	Every day<br>Not every day<br>Not at all<br>I do not know / I do not want to answer this question
	Past smoking status for smokers who currently do not smoke every day	Q2a. In the past, did you smoke cigarettes every day?	Yes<br>No
	Past smoking status	Q2b. In the past, did you smoke cigarettes every day or not every day?	Every day<br>Not every day<br>I do not know / I do not want to answer this question
Intensity of physical addiction to nicotine, differentiation between biological and behavioral addiction		Q3. How many cigarettes do you smoke on average per day or per week?	10 a day or less<br>11-20 a day<br>21-30 a day<br>31 a day or more<br>10 a week or less<br>11-20 a week<br>21-30 a week<br>31 a week or more<br>I do not know / I do not want to answer this question
		Q4. How soon after waking up do you smoke your first cigarette?	Within the first 5 minutes<br>Within the first 6-30 minutes<br>Within the first 31-60 minutes
		Q5. Do you find it difficult to refrain from smoking in places where it is not allowed?	Yes<br>No
		Q6. Can you refrain from smoking even when you are	Yes<br>No

	sick and stay in bed most of the day?	
	Q7. What type of tobacco products do you use most often?	Cigarettes<br>E-cigarettes<br>Other<br>Tobacco heating system<br>Water pipe<br>Pipe<br>Cigar<br>I do not know / I do not want to answer this question
Effectiveness of smoking cessation program	Q8. Since you joined the program, have you had any consultations in the tobacco treatment center?	Yes<br>No<br>I do not know / I do not want to answer this question
	Q9. Since you joined the program, how many times have you tried to quit smoking?	*(provide the number of times)*
	Q10. How long is it since you have quitted smoking?	*(provide the number of months)*
	Q11. Have you used pharmacotherapy to help you quit smoking?	Yes<br>No<br>I do not know / I do not want to answer this question
	Q12. What type of medicine or nicotine replacement therapy have you used?	*(provide the name of the product)*
Motivation for smoking cessation	Q13. What made you quit smoking?	*(provide the reason)*
